# Cooperation Improves Success during Intergroup Competition: An Analysis Using Data from Professional Soccer Tournaments

**DOI:** 10.1371/journal.pone.0136503

**Published:** 2015-08-27

**Authors:** Gwendolyn Kim David, Robbie Stuart Wilson

**Affiliations:** School of Biological Sciences, The University of Queensland, St Lucia, QLD 4072, Australia; Universidad Carlos III de Madrid, SPAIN

## Abstract

The benefit mutually gained by cooperators is considered the ultimate explanation for why cooperation evolved among non-relatives. During intergroup competition, cooperative behaviours within groups that provide a competitive edge over their opposition should be favoured by selection, particularly in lethal human warfare. Aside from forming larger groups, three other ways that individuals within a group can cooperate to improve their chances of gaining a mutual benefit are: (i) greater networking, (ii) contributing more effort, and (iii) dividing labour. Greater cooperation is expected to increase the chances of gaining a group benefit by improving proficiency in the tasks critical to success—yet empirical tests of this prediction using real-world cases are absent. In this study, we used data derived from 12 international and professional soccer competitions to test the predictions that: 1) greater levels of cooperative behaviour are associated with winning group contests, 2) the three forms of cooperation differ in relative importance for winning matches, 3) competition and tournament-type affect the levels of cooperation and shooting proficiency in matches, and 4) greater levels of networking behaviour are associated with increased proficiency in the most critical task linked with winning success in soccer—shooting at goal. Winners were best predicted by higher shooting proficiency, followed by greater frequencies of networking interactions within a team but unexpectedly, fewer networking partners and less division of labour. Although significant variation was detected across competitions and tournament-types, greater levels of networking behaviour were consistently associated with increased proficiency in shooting at goal, which in turn was linked with winning success. This study empirically supports the idea that intergroup competition can favour cooperation among non-relatives.

## Introduction

Why should one cooperate with non-relatives when it imposes a personal cost and benefits others? The benefits mutually gained by cooperators, immediately or in the future, are considered the ultimate explanation for why cooperation evolved among non-relatives [1,2]. Particularly in intergroup competition, the cultural or biological traits that underlie cooperative behaviour within a group that provide a competitive edge over another should be favoured by selection in humans [3,4,5,6]. A study of 625 historical battles between 1600–1973 found numerically larger armies were more likely to be victorious over smaller ones [7], yet they can be harder to coordinate [8]. Forming a larger group than an opponent however, is not the only cooperative behaviour that can provide a competitive advantage during intergroup competition.

There are three main pathways whereby individuals within a group, or as a collective unit, can cooperate to gain a competitive advantage during intergroup competition: (i) greater networking, (ii) contributing more effort and (iii) task specialisation to create effective division of labour within a group. Networking is the maintenance of non-random cooperative interactions between individuals within a group whereby established pathways between critical partners may improve the speed or quality of tasks important to group success. For example, collaboration with particular key individuals has been associated with publication success in academia [9,10] and winning competitive soccer matches [11]. An individual’s contributed effort represents the magnitude of their actions performed for the group and incurs a personal cost, whereby greater contributions by a group should increase the opportunities to successfully complete important tasks. For example in a study on forest commons management in Ethiopia, groups containing individuals that contributed more time to costly forest patrols yielded higher potential crop returns than those that contributed less [12]. Finally, if there are a range of actions required to gain a mutual benefit, division of labour among the individual members within a group is expected to improve proficiency in the chain of multiple tasks [13,14,15]. Division of labour is a well studied cooperative behaviour associated with hunting success in humans and other animals [13,14,15]. Although cooperative behaviours have been associated with group success, our understanding of how cooperation provides a competitive edge during intergroup competition is less clear.

The mechanism whereby cooperation provides a competitive advantage for a group is expected to occur via increases in the success and efficiency in those tasks critical to gaining the shared mutual benefit [13,14,15]. Despite the logic of this idea, empirical tests of this prediction using real-world cases are virtually absent [16,17,18], yet it is an assumption underlying that intergroup competition played a pivotal role in the evolution of human cooperation [3,4,5,6]. Based on this idea we expect that during intergroup competition, successful groups should have greater levels of cooperative behaviours that are associated with higher proficiencies in those tasks critical to gaining the mutually shared benefit. Although this theory has been formulated in the context of the evolution of human cooperation, the logic should equally apply to intergroup competitions that occur within the more artificial construct of human sporting competitions. To empirically test this expectation, we used data derived from six international soccer tournaments (*FIFA* World Cup 2010 and 2014, Copa América 2011, *UEFA* Euro Cup 2012, *CAF* Africa Cup of Nations 2012 and *FIFA* Confederations Cup 2013) and six professional leagues in the 2013/14 season (Australian A-League, English Premier League, Dutch Eredivisie, Italian Serie-A, Russian Premier League and Spanish La Liga) to explore how different cooperative behaviours affect success during intergroup competition. Firstly, a detailed dataset derived from the *FIFA* World Cup 2010 was used to test that: 1) greater levels of cooperative behaviour were associated with winners, and 2) the three forms of cooperation (networking, contributing effort and division of labour) differed in relative importance for winning matches. Since the outcome of soccer matches is based upon goal difference, cooperative behaviour can only contribute indirectly to group success via shooting at goal. Therefore, we then collected data on networking behaviour from 5 other international tournaments and 6 professional leagues to test that: 3) competition and tournament-type effected the levels of cooperation and shooting proficiency in matches, and 4) greater levels of networking behaviour were associated with increased proficiency in the most critical task linked with winning success—shooting at goal.

## Methods

This study was approved by the Ethics Committee at the University of Queensland, whereby the need for informed consent was waived. To test the first two predictions, detailed statistical information from matches that resulted in a win/loss in the *FIFA* World Cup 2010 was published and collected online (www.fifa.com). This dataset contained 48 matches involving 32 teams, and consisted of performance data relating to the skilled execution of tasks involving the ball (clearing, passing, receiving, shooting and tackling) and physical contributions (sprints and kilometres covered), for both individuals and teams (sum total of individuals in a team) in each match. For each skilled performance task (clearing, passing, shooting and tackling), three measures were used; *success* (number of successfully executed actions), *activity* (number of attempted actions) and *efficiency* (successes per action). Note that in the shooting task, a successfully executed action occurred if a shot was made on target but did not necessarily result in a scored goal. For the receiving task, there were only data available for successes. Performance data for individuals were standardised to minutes played. Any individual with less than 30 minutes total participation in a single match (less than 1/3^rd^ of the match) were excluded from the dataset for that specific match because their higher rates of success and activity can exaggerate their contributions relative to other individuals.

To test the third and fourth predictions, a larger sample size was required for the analyses. In addition to the *FIFA* World Cup 2010 dataset, data on team shooting and passing performance in matches that resulted in a win/loss were collected online from five other international tournaments and six professional leagues for a total of 1564 matches: Copa América 2011 (N = 15) (www.ca2011.com), *UEFA* Euro Cup 2012 (N = 24) and *CAF* Africa Cup of Nations 2012 (N = 27) (FourFourTwo STATS ZONE application), *FIFA* Confederations Cup 2013 (N = 13) and *FIFA* World Cup 2014 (N = 50) (www.fifa.com), Australian A-League 2013/14 (N = 104) (www.a-league.com.au), whereas English Premier League 2013/14 (N = 302), Dutch Eredivisie 2013/14 (N = 221), Italian Serie-A 2013/14 (N = 290), Russian Premier League 2013/14 (N = 177) and Spanish La Liga 2013/14 (N = 293) were sourced from an alternative website (www.whoscored.com). A match is 90 minutes long, therefore for any match that went into extra time (an additional 30 minutes = 120 minutes total) data were corrected by multiplying by 0.75 so that these matches would not over-contribute to analyses.

### Cooperative behaviours

Networking behaviour in soccer can be based upon passing between players on the same team as it relies on cooperation among pairs of individuals to deliver and successfully receive the soccer-ball. Using social network analysis, which quantifies the interactions among individuals within a group, the level of networking behaviour via passing cooperation among pairs of players within a team was quantified using measures of connectedness. Two measures of connectedness [19] were calculated for each player (node) based on the successful passes executed between pairs of players (edges): degree and strength. The degree of an individual refers to the number of other players they cooperated with during a match via successful passes (either delivering or receiving the soccer-ball). The strength of an individual refers to the sum of all the cooperative interactions via successful passes that were executed with all other players in a team (sum of edge weights). A team’s degree and strength were calculated by taking the average across individual player values for a team in each match [19].

Contributing effort is a cooperative behaviour in soccer because every action a player performs in a match contributes to his whole team’s likelihood of a shared mutual benefit (ie. match-win) but comes at a personal energetic cost and injury risk. For each team in every match, three measures of contributing effort were calculated: (i) the total number of skilled actions attempted, (ii) the number of sprints performed, and (iii) the total number of kilometres covered.

Division of labour (DoL) is a form of cooperation as it relies on individuals within a group to coordinate the range of activities required for a collective aim associated with gaining a shared mutual benefit. Previous studies refer to two types of individuals within a group expressing DoL: specialists (attempting only one type of the group’s multiple tasks) and generalists (attempting all of a group’s multiple tasks) [15]. However, these categories only refer to the extreme forms of DoL, and it is more likely that DoL is expressed along a continuum between specialists and generalists. For example, individuals that attempt only a subset of a group’s total range of tasks could be considered as partial specialists. A group’s DoL can then vary from complete DoL (all individuals are specialists), to incomplete DoL (co-existence of specialists, partial specialists and generalists) to no DoL (all individuals are generalists). In our study, we calculated a team’s level of DoL to reflect such a continuum between specialists and generalists. We first determined the total number of skilled tasks (clearing, passing, receiving, shooting and tackling) each player participated in during a match (ie. range 0–5)—referred to as an individual’s DoL score. This value was summed up across all players within the team—referred to as a team’s DoL score. Then, the theoretical maximum value for a team’s DoL score was calculated (number of tasks multiplied by the number of individuals in the team). To make it more intuitive to interpret, a team’s DoL score was therefore calculated as: DoL = 1- (sum of individual DoL scores/theoretical maximum of team DoL). Here, a value of zero reflects no DoL as all players in a team performs each of the five skilled tasks, whereas a value close to 1 reflects complete DoL.

### Analyses

To test the first two predictions, generalised linear models (GLMs) were used to avoid the risk of inflated type I error with multiple paired t-test comparisons. To assess the relative importance of six measures of cooperative behaviour (degree, strength, skilled actions attempted, sprints performed, kilometres covered and DoL) and shooting proficiency (success, efficiency or activity) for winning matches in the *FIFA* World Cup 2010, a focal dataset was created from the standardised dataset, whereby for each match only one team’s performance data was analysed to avoid pseudo-replication (maintaining an even number of winning and losing teams). To assess the probability of winning a match in the *FIFA* World Cup 2010 (n = 48), the GLM (family = Binomial) was fitted with seven predictor variables without interactions, and a binary response variable (win, loss).

To test whether greater levels of networking behaviour were indirectly associated with winning success by enhancing proficiency of shooting at goal, structural equation modelling (SEM) was used following tests for significant competition and tournament-type effects. Since a sample size of less than 100 in SEM is considered small [20], the *FIFA* World Cup 2010 dataset was combined with data from 11 other competitions: Copa América 2011, *UEFA* Euro Cup 2012, *UEFA* Africa Cup of Nations 2012, *FIFA* Confederations Cup 2013, *FIFA* World Cup 2014, Australian A-League 2013/14, English Premier League 2013/14, Dutch Eredivisie 2013/14, Italian Serie-A 2013/14, Russian Premier League 2013/14 and Spanish La Liga 2013/14 (n = 1564). Instead of mean player strength within the team however, team total ‘passing success’ was used as the measure of networking behaviour. For each match in the dataset, one team was randomly assigned as the focal team, maintaining an even number of winning and losing teams. To test for significant effects of competition (12 factor levels: individual competitions) and tournament type (2 factor levels: international knock-out phases, professional league rounds) on team shooting and networking, analyses of variance (ANOVAs) and t-tests were calculated. To analyse the indirect association of focal and opposition networking behaviour with winning success via shooting proficiency, SEM’s were calculated using the software program *MPlus* v6.12 [21] on standardised data. Since ‘winning success’ was a binary response variable (win or loss), a robust weighted least-squares estimator (WLSMV) using probit regression was used [21] whereby the predictor variables were treated as continuous and their values centred around the grand-mean: focal-team shooting success, focal-team shooting activity, focal-team and opposition-team networking behaviour. Since the outcome of soccer matches is based upon goal difference, cooperative behaviour can only contribute indirectly to winning matches via shooting attempts successfully aimed at the goal. As such, only successful shots on target could directly contribute to winning whereas total shooting activity and networking behaviour contribute indirectly. Goodness of fit was based on the comparative fit index (CFI), whereby a value greater than 0.95 indicates a good model fit [22].

## Results

Although higher shooting proficiency strongly contributed to winning in the *FIFA* World Cup 2010, only some cooperative behaviours were positively associated with winning (Table 1). Shooting proficiency was consistently greater in winning compared to losing teams (successful shots, winner = 7, loser = 4; total number of shots, winner = 16, loser = 13; shooting efficiency, winner = 0.4, loser = 0.3). Compared to those that lost, winning teams contained players with higher frequencies of networking interactions on average (winner = 71, loser = 60), but only slightly fewer passing partners. Unexpectedly teams with less division of labour were more likely to win matches, whereas the contribution of effort had no significant effect on winning. Therefore, the probability of winning a match in the *FIFA* World Cup 2010 was best predicted by higher shooting proficiency, more networking interactions and less division of labour.

**Table 1:** Contribution of cooperative behaviours and shooting proficiency to the probability of winning matches in the *FIFA* World Cup 2010.

Variables	Shooting variable											
	Success				Activity				Efficiency			
	*Est*.	*SE*	*Z*	*P*	*Est*.	*SE*	*Z*	*P*	*Est*.	*SE*	*Z*	*P*
(Intercept)	0.3	0.4	0.4	0.512	0.3	0.4	0.4	0.507	0.3	0.4	0.8	0.378
Shooting	2.0	0.7	12.1	**0.001**	1.4	0.6	6.9	**0.009**	1.4	0.7	5.4	**0.020**
Networking												
strength	6.7	3.6	4.6	**0.033**	5.8	3.2	3.9	**0.047**	4.6	3.0	2.7	0.099
degree	-1.6	0.8	4.5	**0.033**	-1.5	0.7	4.9	**0.027**	-1.2	0.7	2.8	0.097
Contributing effort												
skilled attempts	-5.1	3.4	2.6	0.109	-4.0	3.1	1.9	0.170	-2.8	2.9	1.0	0.313
sprints	-0.1	0.7	0.0	0.920	-0.1	0.6	0.0	0.886	0.5	0.6	0.7	0.419
*km* covered	1.0	0.8	1.7	0.192	1.1	0.7	2.3	0.134	0.1	0.7	0.0	0.886
Division of labour	-1.2	0.5	7.3	**0.007**	-1.1	0.5	7.1	**0.008**	-1.0	0.4	7.9	**0.005**

Generalised linear models (family = Binomial) assessed the probability of winning matches (N = 48); seven predictor variables fitted with no interactions and a binary response variable. ***Est***. (Estimate), ***SE*** (Standard Error), ***Z*** (Z-value), ***P*** (P-value); bold type highlights significant effects.

**Fig 1:**
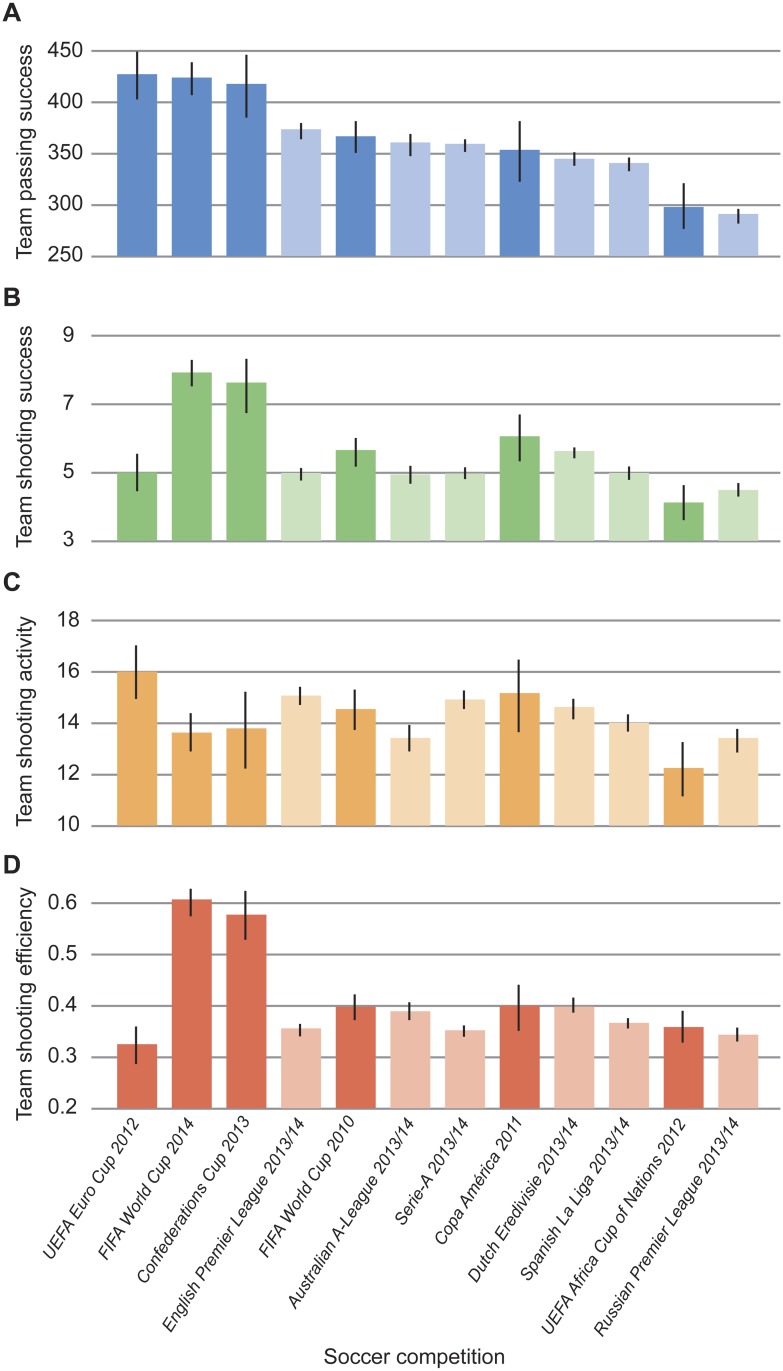
Competition and tournament-type effects on networking behaviour and shooting proficiency. Average level of (*A*) networking behaviour, based on total number of successful passes, (*B*) shooting success, (*C*) shooting activity and (*D*) shooting efficiency of teams within matches (*N* = 1564). Dark and light shaded bars denote international tournaments and professional leagues, respectively. Standard error bars are shown.

Competition and tournament-type affected the levels of networking behaviour and shooting proficiency in matches (Fig 1). Competition significantly affected team passing success (*F* = 10.34, *P* < 0.001) and there were more successful passes in international tournaments compared to professional leagues (successful passes: international = 382, professional = 347, *t* = 3.61, *P* < 0.001). Shooting proficiency varied significantly across competitions (shooting success, *F* = 7.75, *P* < 0.001; shooting activity, *F* = 2.62, *P* = 0.003; shooting efficiency, *F* = 14.17, *P* < 0.001). Whilst shooting success and efficiency were higher in international tournaments compared to professional leagues (successful shots: international = 6, professional = 5, *t* = 4.08, *P* < 0.001; shooting efficiency: international = 0.43, professional = 0.35, *t* = 6.01, *P* < 0.001), the total number of shots in a match did not differ.

**Fig 2:**
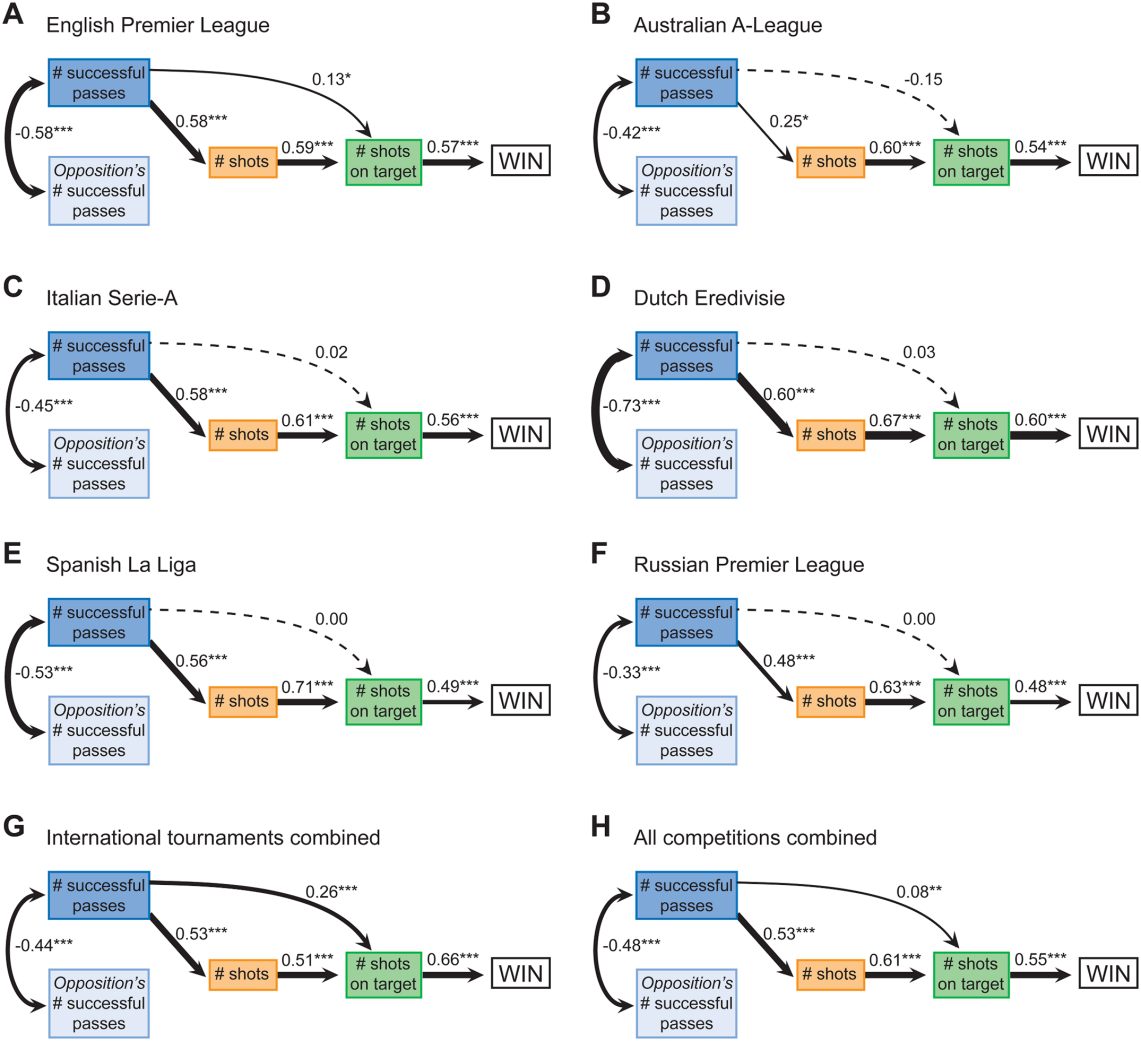
Relationship between cooperation, proficiency and victory in soccer competitions. Structural equation models (SEM) illustrate the indirect association between a team’s level of cooperation and winning matches, via proficiencies in the critical task of shooting in: (*A-F*) professional leagues, (*G*) international tournaments combined and (*H*) all competitions combined. Coefficients presented are standardised estimates based on probit regression, whereby interpretation can only be made on sign and significance (*P < 0.05, **P < 0.01, ***P < 0.001). Dotted line denotes insignificant relationships. For SEMs, comparative fit index (CFI) values greater than 0.95 indicate good model fit [22]: CFI (*A-F*) = 0.96, 1.00, 0.95, 0.91, 0.98, 0.99, 0.86 and 0.96 (note model *G* has a low CFI and should be interpreted with caution).

Greater levels of networking behaviour were consistently associated with increased proficiency in shooting at goal, which in turn was linked with winning success, however this pattern significantly varied across competitions (Fig 2). More successful passes were consistently associated with a decrease in the opposition’s number of networking interactions, a greater number of shooting attempts and (indirectly) winning success. In the Australian A-League however, the positive relationship between successful passes and number of shooting attempts is weaker relative to all other competitions. Successful passes were only positively associated with a greater number of shots on target in the EPL, international tournaments and when all the data were combined (Fig 2*A*, 2*G* and 2*F*). Overall, greater levels of networking behaviour were associated with increased proficiency in the most critical task linked with winning success—shooting at goal.

## Discussion

Success in professional soccer tournaments was associated with greater levels of networking by increasing proficiency in the critical task of shooting, empirically supporting the idea that cooperative behaviour can improve the chance of a shared beneficial gain for cooperators [1,2]. Greater frequencies of cooperative interactions through passing, but fewer networking partners, were associated with winning success which supports that interactions with certain key players may be more influential than others within a team [11]. Some individuals contribute more than others to their group’s success which may be associated with the opposition quality, positional roles, personal capacities, injury or motivation [9,11]. Not all cooperative behaviours increased the probability of winning soccer matches highlighting that particular forms of cooperation may be more important for gaining the mutual shared benefit than others [9].

Greater frequencies of cooperative interactions were associated with winning matches in professional soccer tournaments by increasing proficiency in shooting—the most critical task linked with group success. This result suggests that greater levels of networking interactions by winning teams compared to their losing opponents create more opportunities for players to shoot, and in some competitions enhance shooting success. The winning teams could also possess higher-quality shooters, more skilled players, better timing of executed actions, superior defence or a combination of all these factors. Understanding how these factors vary across teams and competitions, and in turn contribute to the relationship between cooperative interactions and proficiency in shooting, would be an interesting avenue for future studies.

Teams that contributed more effort than their opposition did not win more matches. As such, executing a greater level of behaviour than needed to gain the disputed resource may impose extra energetic costs and risk of injury to players in sport, or may unnecessarily increase the chance of death for soldiers in the case of warfare. This result highlights that focusing on the quality of collective actions of a group, rather than just mere quantity, may be more beneficial for success in intergroup competition.

When individuals within a group perform only one specific task which is complementary to others that contribute to a common goal, enhanced effectiveness is expected through the division of labour [13,14,15]. We found no evidence that greater division of labour contributed to winning soccer matches, but rather a negative association was evident. As such, our study does not empirically support the theoretical prediction that greater division of labour increases the likelihood of a beneficial gain [13,14]. Instead, the probability of winning increased as division of labour decreased suggesting that soccer teams may benefit when all players perform most tasks as this may improve scoring opportunities and reduce predictability of play. Within a soccer team however, players take on positional roles which may lead to certain individuals performing most of their team’s actions in a particular skilled task relative to others. For example, a higher proportion of a team’s shooting attempts may be performed by forwards compared to individuals in other positional roles. Such positional roles were not reflected in our measure of division of labour, and in turn may increase the predictability of a player’s actions. In future work, taking into account how often an individual performs a particular task *relative* to others in their group, rather than the strict measure of does or does not perform, may be a more informative in studies investigating the division of labour. Furthermore, this study demonstrated that greater division of labour may not be beneficial during intergroup competition.

The negative relationship between the competing teams’ level of networking could be largely explained by there being only one soccer-ball, of which a team must be in possession to carry out the behaviour. The maintenance of cooperation during intergroup competition may therefore reflect reduced opportunity to express cooperative behaviour imposed by the abilities of the competing group, rather than players within a team choosing to be uncooperative. This is important when considering the vast literature investigating the problem of free-riders in human cooperation [23]; the propensity of unrelated individuals to cooperate within a group may also be associated with opportunity or personal abilities rather than just an active choice to be uncooperative.

Our work has two important outcomes for the management of soccer teams in professional leagues and international tournaments. First, greater activity in physical tasks does not translate into greater team success. A greater number of sprints by individuals in a team, amount of ball-related activities, or distance covered had no association with the probability of winning matches. This does not mean that these traits are unimportant but it does mean that they do not separate the winning from the losing teams. Each team is likely to require a certain amount of physical effort to be competitive within a game but this does not determine the probability of winning. Rather, more passing interactions between particular players are likely to create better scoring opportunities to win matches than simply performing any action as often as possible. Secondly, the probability of winning increased as division of labour decreased suggesting that soccer teams may benefit when all players perform most tasks–empirically supporting the influential tactical theory “Total Football” posed by the Dutch football club *Ajax*. In competitive soccer therefore, less division of labour may be particularly important as it could decrease a team’s predictability of play and may allow *any* player to take advantage of opportunistic scoring situations.

In this study of professional soccer tournaments, we provide empirical support that increased cooperation among non-relatives is associated with the improved chances of gaining a mutually shared benefit during intergroup competition by enhancing proficiency in a critical task [4,14]. This study empirically shows that greater levels of certain cooperative behaviours within a group can provide a competitive edge in intergroup competition, and thereby supports the idea that inter-group conflict most likely played an important role in favouring cooperation among non-relatives during human evolution [3,4,5,6].
